# Switchback RNA

**DOI:** 10.1021/acschembio.4c00518

**Published:** 2024-09-24

**Authors:** Bharath Raj Madhanagopal, Hannah Talbot, Arlin Rodriguez, Arun Richard Chandrasekaran

**Affiliations:** † The RNA Institute, University at Albany, State University of New York, Albany, New York 12222, United States; ‡ Department of Nanoscale Science and Engineering, University at Albany, State University of New York, Albany, New York 12222, United States

## Abstract

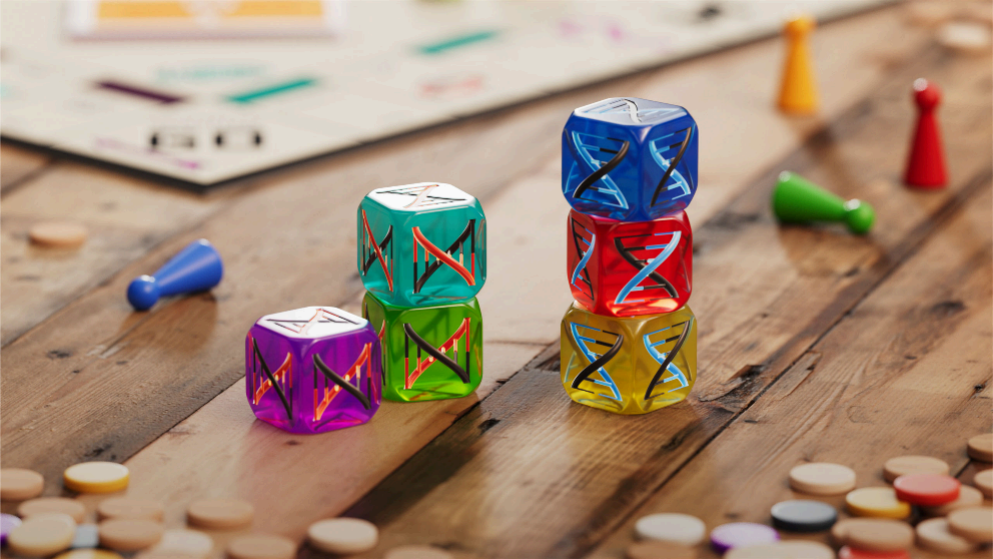

Intricately designed DNA and RNA motifs guide the assembly of robust and functional nucleic acid nanostructures. In this work, we present a globally left-handed RNA motif with two parallel strands called switchback RNA and report its assembly, biophysical, and biochemical characterization. Switchback RNA can be assembled in buffers without Mg^2+^, with improved thermal stability in buffers containing Mg^2+^, Na^+^, or K^+^. Differences in the binding of small molecules to switchback RNA and conventional RNA indicate design-based approaches for small molecule loading on RNA nanostructures. Further, the differential affinity of the two component strands in switchback or conventional duplex conformations allows for toehold-less strand displacement. Enzyme studies showed that the switchback and conventional RNA structures have similar levels of nuclease resistance. These results provide insights for employing switchback RNA as a structural motif in RNA nanotechnology. Our observation that RNA strands with switchback complementarity can form stable complexes at low magnesium concentrations encourages studies into the potential occurrence of switchback RNA in nature.

The design, assembly, and characterization of DNA and RNA motifs are key to the bottom-up construction of nucleic acid nanostructures. While DNA-based nanostructures are more prevalent and well-studied, there is increasing interest in the development of RNA-based nanostructures.^1,2^ The conformationally dynamic and diverse nature of RNA allows it to form various structural motifs.^3^ Inspired by these, several RNA motifs and their assemblies have been created, including those that are unique to RNA, such as RNA kissing loops,^4^ C-loops,^5^ packaging RNA of phi29 bacteriophage,^6^ kink-turn motifs,^7^ and multiarm junctions.^8^ Synthetic RNA motifs that are mimics of DNA motifs and nanostructures have also been developed, such as the RNA double crossover motif used in RNA nanotube assembly,^9^ paranemic crossover RNA structures used for modular assembly,^10,11^ and 3-point-star and 4-point-star tiles that form hybrid RNA:DNA two-dimensional arrays.^12^ Other examples of RNA nanostructures include cubes,^13^ polyhedra,^14^ hybrid RNA:DNA tensegrity triangles,^15^ as well as origami structures that are all-RNA^16^ or hybrid origami structures containing an RNA scaffold and DNA staple strands.^17−19^ Some of these RNA nanostructures have been used to control gene expression in yeast,^20^ trap and release fluorescent aptamers,^21^ in bioimaging,^22^ and drug delivery.^23,24^

In this work, we present switchback RNA and switchback RNA:DNA hybrid motifs that are based on our recent work on switchback DNA.^25^ We provide a detailed comparison of the biophysical and biochemical properties of switchback RNA with the conventional duplex RNA. The switchback motif is assembled from two strands and contains a series of half-turns aligned laterally, where the helical axis of the half-turn domains is perpendicular to the axis of the full structure (Figure 1a).^26−28^ The switching back of the backbone after each half-turn results in a structure that is a globally left-handed helix with the two strands arranged parallel to each other. In switchback RNA, each half-turn domain consists of six base pairs, with the two strands being complementary in the switchback sense.

**Figure 1:**
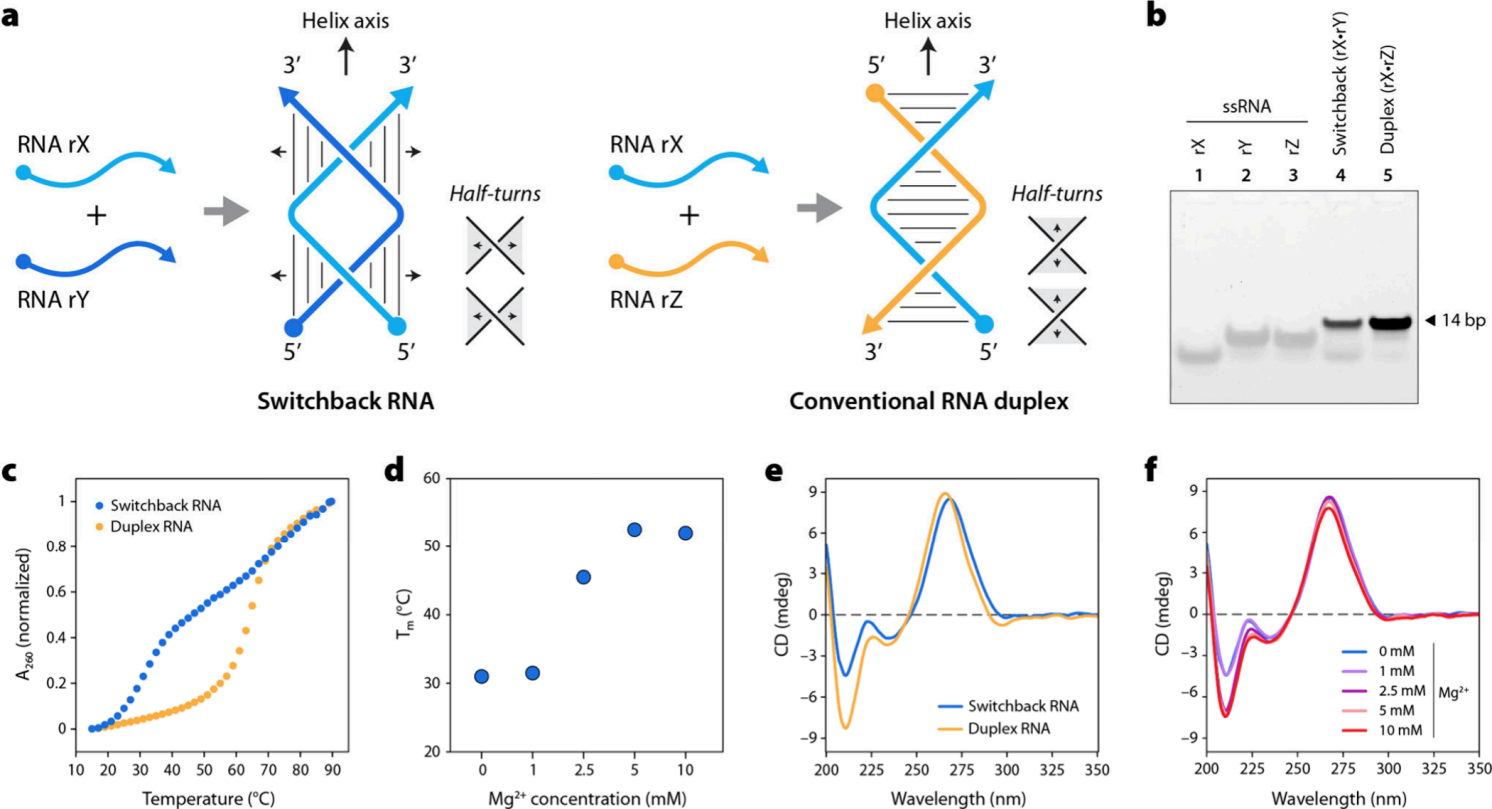
Design and assembly of switchback RNA. (a) Schematic representation of switchback RNA and conventional RNA duplex. (b) Nondenaturing gel image showing the formation of switchback RNA in Mg^2+^-free TAE buffer. (c) Comparison of the UV-thermal melting curves of switchback RNA and conventional RNA duplex in TAE buffer. (d) Effect of Mg^2+^ on the melting temperature of switchback RNA. (e) Comparison of the circular dichroism (CD) spectra of switchback RNA and conventional RNA duplex in TAE buffer. (f) Mg^2+^ concentration-dependent change in the CD spectra of switchback RNA.

To test the formation of switchback RNA, we used the sequences we recently reported for the assembly of switchback DNA molecules (Figure S1 and Table S1).^25^ We annealed the two component RNA strands (denoted rX and rY) in 1× tris-acetate-EDTA (TAE) buffer and validated self-assembly using nondenaturing polyacrylamide gel electrophoresis (PAGE) (Figure 1b). As a control, we used a duplex complement (denoted rZ) to form a conventional duplex with strand rX (complex rX·rZ). We use the term “switchback complement” for the strand rY that can form a switchback structure with rX, and the term “duplex complement” for the strand rZ that forms a conventional A-form duplex. Unlike switchback DNA which required Mg^2+^ to form,^25^ switchback RNA assembled in buffer without any Mg^2+^. This was confirmed by the similar migration of the two-stranded switchback RNA (Figure 1b, lane 4) and the conventional RNA duplex (Figure 1b, lane 5). Interestingly, when the RNA strands rX and rY were annealed in Mg^2+^-containing buffer, the complex (rX·rY) showed a single band that migrated slower compared to the conventional duplex (Figure S2), indicating a larger complex or Mg^2+^-bound switchback RNA with different electrophoretic properties. Further, the formation of this complex was influenced by the concentration of magnesium and appeared only in the buffer containing >2.5 mM Mg^2+^. To distinguish the structures formed by complex (rX·rY) in the presence and absence of Mg^2+^, we compared their mobilities as a function of polyacrylamide concentration using a Ferguson plot, where the slope provides an estimate of the frictional coefficient of the structures (Figure S3). The (rX·rY) and (rX·rZ) complexes exhibited similar retardation, regardless of the gel percentage, in Mg^2+^ free buffer. The slope of the (rX·rY) complex varied from that of the conventional RNA duplex (rX·rZ) in the Mg^2+^-containing buffer, indicating that the switchback RNA undergoes a structural transformation in the presence of Mg^2+^ ions.

We then analyzed the thermal stability of switchback RNA and obtained a melting temperature (*T*_m_) of ∼31 °C in a Mg^2+^-free buffer (Figure 1c). The presence of 1 mM Mg^2+^ in the annealing buffer did not affect the *T*_m_ but showed a Mg^2+^ concentration-dependent increase to 52 °C at 10 mM Mg^2+^ (Figure 1d and Figure S4). The conventional duplex showed a relatively higher *T*_m_ at these concentrations of Mg^2+^ (Figure S4 and Table S2). The formation and thermal stability of switchback RNA at 1 mM and 2.5 mM Mg^2+^ is significantly higher compared to switchback DNA in similar Mg^2+^ concentrations.^25^ We then compared the conformations of (rX·rY) and (rX·rZ) complexes using circular dichroism (CD) spectroscopy. Switchback RNA assembled in solutions containing no Mg^2+^ and 1 mM Mg^2+^ exhibited a strong positive band at 265–270 nm and a negative band at 210 nm, which are characteristic of double-stranded A-RNA^29^ (Figure 1e-f). This confirmed that the underlying half-turns in switchback RNA are typical right-handed A-RNA helices despite the overall left-handedness of the switchback structure. In the absence of Mg^2+^, the amplitude of the negative band at 210 nm was less than that of the conventional duplex, whereas in Mg^2+^ concentrations above 2.5 mM, the amplitude of the band increased, and the CD spectra of switchback RNA closely resembled those of conventional RNA duplex (Figure 1f and Figure S5). These results suggest that Mg^2+^ induces conformational changes in switchback RNA at higher concentrations. It is of note that we previously observed a similar trend with DNA where the CD spectra of both switchback DNA and conventional duplex showed the typical CD signature of B-DNA.^25^ To test whether other metal ions provide enhanced thermal stability to switchback RNA without changing its electrophoretic properties, we annealed the structures in TAE buffer containing 25 mM Na^+^ or K^+^. In these cases, the switchback RNA band migrated similar to the conventional duplex (Figure S6). We performed UV melting studies for structures assembled in TAE buffer containing 5–20 mM Na^+^ or K^+^ and observed a marginal increase in *T*_m_ (Figure S7 and Table S3).

In our previous work, we showed that switchback DNA can be used for toehold-less strand displacement reactions.^25^ That is, the addition of a duplex complement displaced a switchback complement from a switchback DNA structure, resulting in a conventional duplex. Here, we first tested the preference of a sequence to pair with its switchback or duplex complement during assembly (Figure 2a and Figure S8). For this experiment, we used a longer rZ strand (rZ_L_) with two additional U residues at the 5′ and 3′ ends (Figure S1) to distinguish the bands corresponding to switchback RNA and conventional duplex in the gel and performed these experiments in a Mg^2+^-free buffer. We annealed strand rX with different ratios of the switchback complement (rY) and duplex complement (rZ_L_) and visualized the resulting complexes using nondenaturing PAGE. When rX is present only with rY or rZ_L_, it forms a switchback RNA (Figure 2a, lane 1) or conventional duplex (Figure 2a, lane 5), respectively. As the ratio of the duplex complement increased, the population of the conventional duplex in the solution increased, with 80% duplex formation when all three strands were present in equal amounts (lane 3). Next, we tested whether a duplex complement could displace a switchback complement from a preassembled switchback RNA. For this, we first assembled the switchback RNA (rX·rY) and incubated it with different ratios of the duplex complement rZ_L_. Addition of rZ_L_ led to a complete conversion of the switchback RNA (rX·rY) into the conventional duplex (rX· rZ_L_) (Figure 2b and Figure S8). However, the addition of switchback complement rY to the conventional duplex did not lead to the formation of switchback RNA (Figure 2c and Figure S8). The ability of a duplex complement to displace a switchback complement from a preassembled switchback RNA structure allows toehold-less strand displacement that is based on the affinity of strands toward a structure in contrast to the purely sequence-controlled toehold-mediated strand displacement.

**Figure 2:**
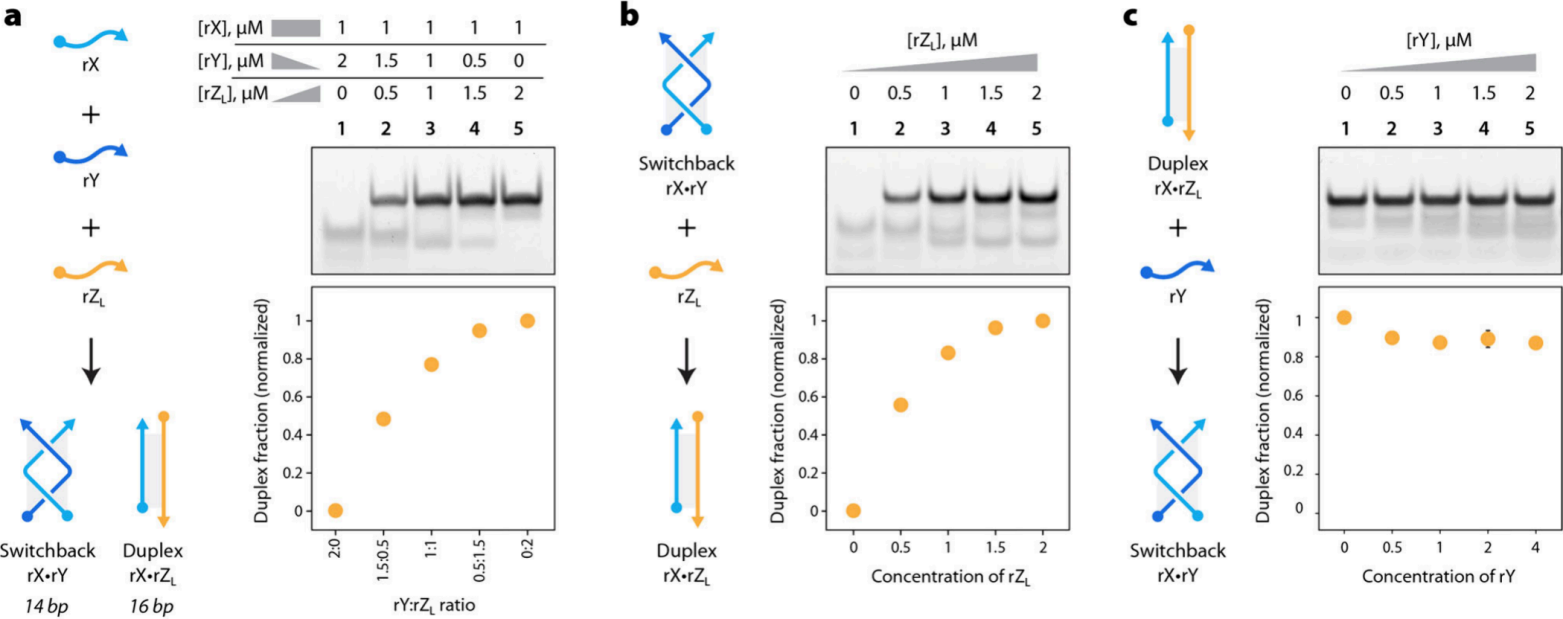
Strand competition and displacement between switchback RNA and conventional duplex. (a) Competition between switchback complement (rY) and a duplex complement (rZ_L_) to bind to rX. (b) A duplex complement (rZ_L_) displaces a switchback complement (rY) from a preassembled switchback RNA. (c) Addition of switchback complement (rY) to a preformed duplex does not displace the duplex complement.

We then analyzed whether the interaction of small molecules is different in switchback RNA and conventional RNA duplexes. We added different concentrations of ethidium bromide (EBr) to switchback RNA and conventional RNA duplex and measured the enhanced fluorescence of the RNA-bound EBr at 600 nm in 1× TAE without Mg^2+^ (Figure 3a-b and Figure S9). The fluorescence of EBr was enhanced more with conventional duplex than with switchback RNA, similar to the trend we observed with switchback DNA and its corresponding DNA duplex.^25^ We then studied the effect of Mg^2+^ on the enhancement of EBr fluorescence on binding to switchback RNA and conventional RNA duplex (Figure 3c and Figure S10). We observed a Mg^2+^-dependent reduction of fluorescence in both structures (at an EBr concentration of 30 μM). While the conventional RNA duplex showed a higher signal in the 0–2.5 mM Mg^2+^ range, the signal was higher for switchback RNA when the concentration of Mg^2+^ in the buffer was 5–10 mM. We then performed similar experiments with the known RNA-binding dye thiazole orange^30^ and observed similar trends in dye binding to switchback RNA and conventional RNA duplex (Figure 3d-f and Figure S11–12).

**Figure 3:**
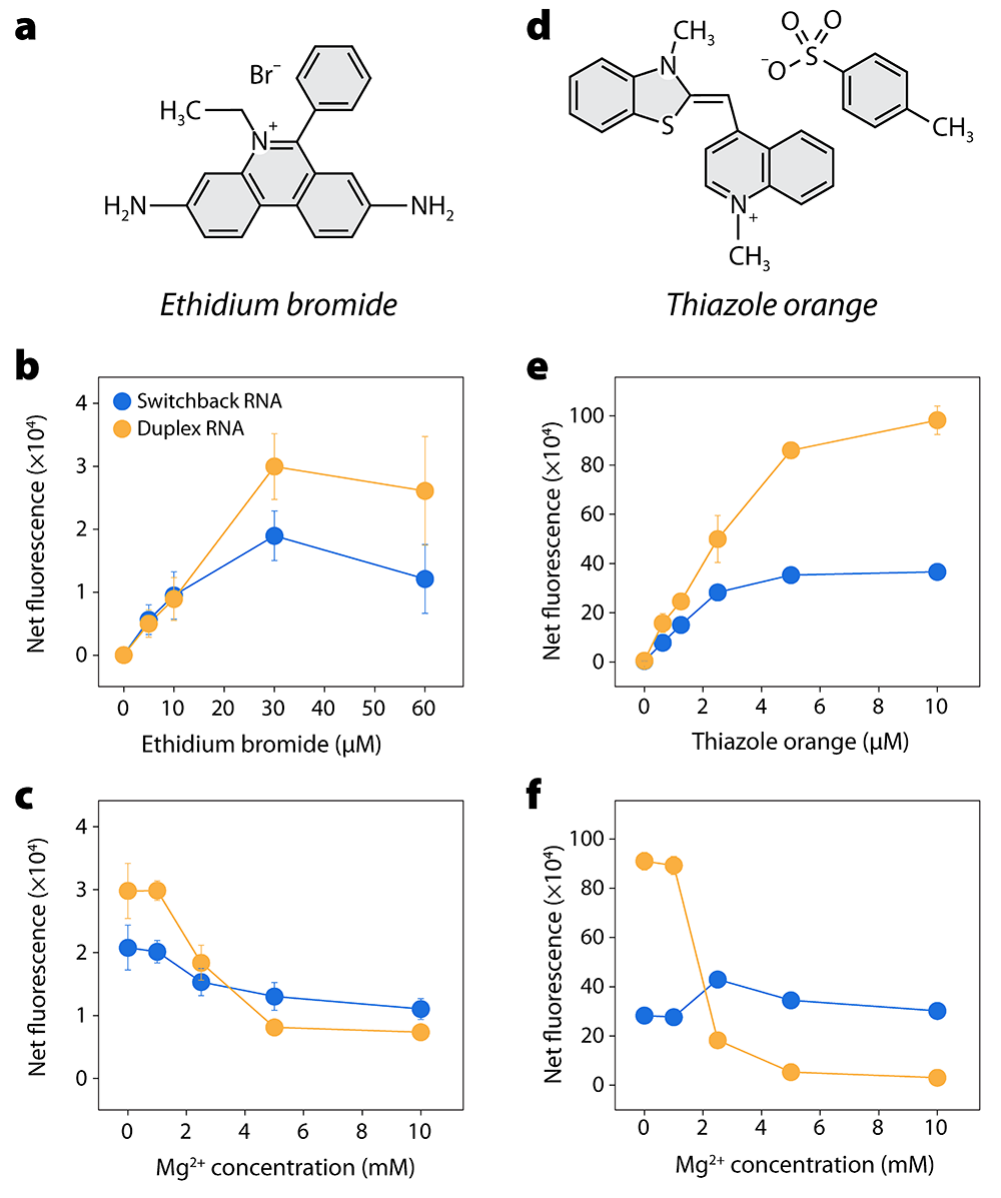
Small molecule binding to switchback RNA. (a) Chemical structure of ethidium bromide. (b) Fluorescence enhancement of ethidium bromide when bound to switchback RNA and conventional duplex. (c) Effect of Mg^2+^ on the fluorescence of ethidium bromide bound to switchback RNA and conventional duplex. (d) Chemical structure of thiazole orange. (b) Fluorescence enhancement of thiazole orange when bound to switchback RNA and conventional duplex. (c) Effect of Mg^2+^ on the fluorescence of thiazole orange bound to switchback RNA and conventional duplex.

Next, we compared the biostability of switchback RNA and conventional RNA duplex. We incubated the annealed structures in different amounts of RNase III at 37 °C for 30 min, analyzed the samples on a nondenaturing PAGE, and quantified the reduction in the intensity of the band corresponding to the structures (Figure 4a and Figure S13). We observed that RNase III degraded switchback RNA and conventional duplex to the same extent, with ∼70% degraded with 20 mU RNase III. We then tested the nuclease resistance of an RNA:DNA hybrid switchback and a conventional RNA:DNA hybrid duplex. We first assembled the hybrid switchback (dX.rY and rX.dY) and validated assembly using nondenaturing PAGE (Figure S14). We then incubated the switchback hybrid and conventional hybrid duplex at 37 °C with RNase H, an enzyme that selectively cleaves the RNA strand in an RNA:DNA hybrid (Figure 4b and Figure S15). Again, we observed that RNase H degraded switchback and duplex RNA:DNA hybrids to the same extent. The length and sequence of the strands used to construct the switchback structures can potentially affect their enzymatic stability. Future studies with larger complexes and diverse sequences may help establish the role of these structural features on the nuclease resistance of switchback RNA and switchback RNA:DNA hybrids. Our current results indicate that switchback RNA can be as robust as conventional RNA to create larger RNA assemblies.

**Figure 4:**
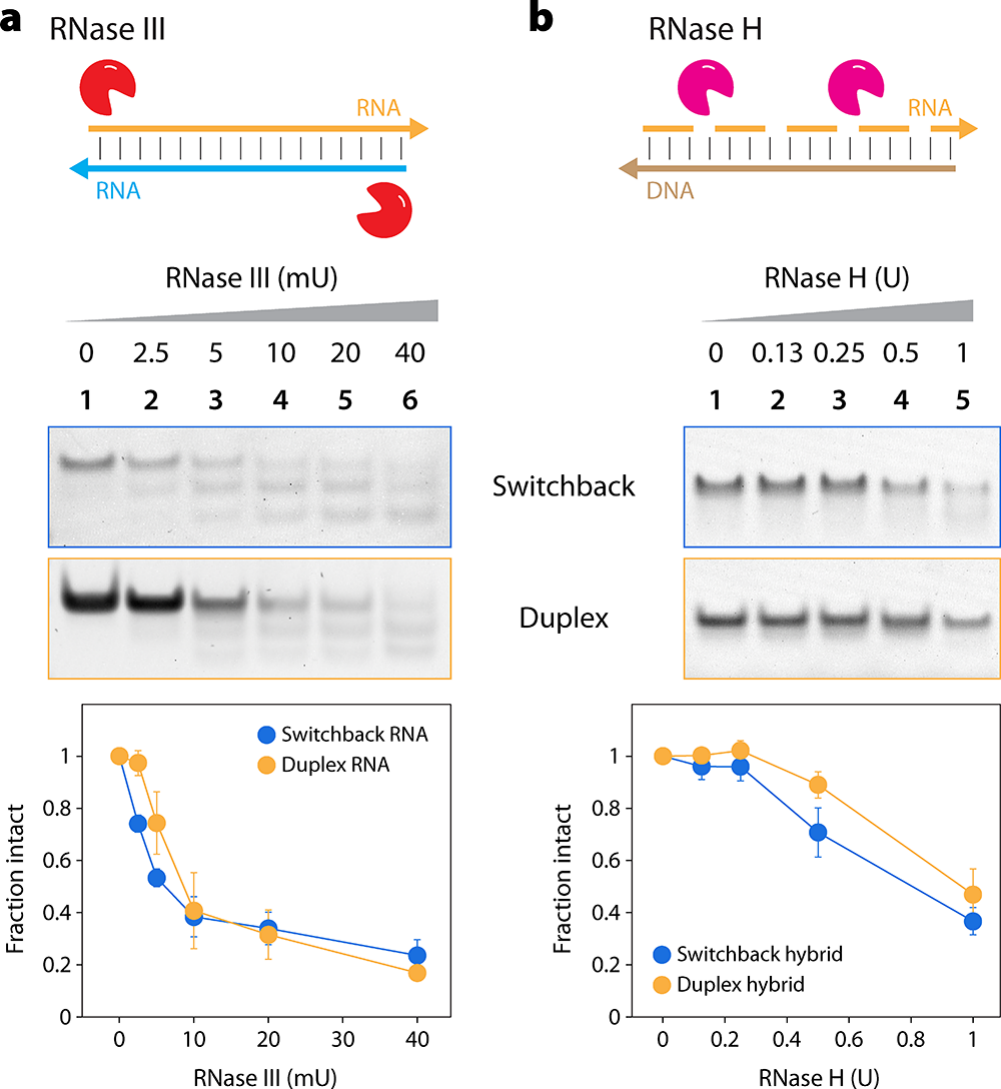
Nuclease resistance of switchback RNA. (a) Degradation of switchback RNA and conventional duplex when treated with different amounts of RNase III. (b) Degradation of switchback RNA:DNA hybrid and conventional hybrid when treated with different amounts of RNase H.

In summary, our work presents the assembly and characteristics of switchback RNA and hybrid switchback RNA:DNA structures. Conformationally, the half-turn domains in the switchback RNA were in the A-form, while the half-turn domains in the switchback DNA were in the B-form. Although switchback RNA and switchback DNA are conformationally distinct structures, their intercalator binding properties were similar. Additionally, the binding of small molecules to switchback RNA was influenced by the concentration of Mg^2+^ in the buffer. The differential binding of small molecules to switchback RNA and conventional duplexes would be useful in the design of drug delivery vehicles where loading is affected by the interaction of small molecules to nucleic acid nanostructures. Switchback RNA is amenable to strand displacement with a duplex complement, obviating the need for toeholds in the design of strand displacement reactions, a characteristic that can also be expanded into higher-order structural reconfigurations.^31^ Switchback RNA exhibited higher thermal stability than switchback DNA. Typically, RNA duplexes are thermally more stable than DNA.^32^ Therefore, this observation is not surprising. However, the dependence of Mg^2+^ to form a switchback structure, which was exhibited by DNA, was not seen with RNA. We had previously hypothesized the potential biological relevance of switchback DNA in short tandem repeats.^25^ The formation of switchback RNA in Mg^2+^-free conditions highlights the need to explore the possible existence of switchback interactions in nature and examine the potential relevance of switchback RNA in biology.^25^ Overall, switchback RNA opens up new avenues in RNA nanotechnology and can be designed into RNA nanostructures for use in biology and medicine.

## Supporting Information Available

Experimental procedures, sequences used, full images of electrophoresis gels, and additional fluorescence and CD spectra (PDF)
