# Supplementary material for: Switchback RNA
Source: ACS Chem Biol. 2024 Sep 24;19(12):2394–8. doi: 10.1021/acschembio.4c00518 (PMC11667664; doi:10.1021/acschembio.4c00518)
Supplement: Supplementary file 1 — cb4c00518_si_001.pdf [file cb4c00518_si_001.pdf]

Supporting Information

Switchback RNA

Bharath Raj Madhanagopal,[1] Hannah Talbot,[1] Arlin Rodriguez,[1] and Arun Richard Chandrasekaran[1,2,*]

[1]The RNA Institute, University at Albany, State University of New York, New York, NY, USA.

[2]Department of Nanoscale Science and Engineering, University at Albany, State University of New York, New York, NY, USA.

*Correspondence: arun@albany.edu

MATERIALS AND METHODS

Preparation of RNA and DNA-RNA hybrid complexes

All the oligonucleotides were purchased from Integrated DNA Technologies (IDT) with standard desalting and were used without further purification. The stock solutions of oligonucleotides were prepared in nuclease-free water, and their concentrations were estimated using Nanodrop 2000 spectrophotometer (Thermo Scientific) by measuring the absorbance of the oligonucleotide solutions at 260 nm. The RNA, DNA, and RNA-DNA hybrid complexes were prepared in tris-acetate EDTA buffer containing 40 mM Tris base (pH 8.0), 20 mM acetic acid and 2 mM EDTA (1× TAE). Magnesium acetate (12.5 mM in the final solution) was added to the buffer to prepare DNA and RNA:DNA hybrids. Oligonucleotide mixtures were annealed in a thermal cycler with the following steps: 95 °C for 5 minutes, 65 °C for 30 minutes, 50 °C for 30 minutes, 37 °C for 30 minutes, 22 °C for 20 minutes, and 4 °C for 2 hours. The solutions were stored at 4 °C.

Gel electrophoresis, imaging, and analysis

Polyacrylamide gels were prepared using 19:1 acrylamide solution (National Diagnostics) in 1× TAE or 1× TAE-Mg^{2+} buffer, and the gels were run using the respective buffers at 4 °C. For samples annealed in Na^+ and K^+, gels were run in the 1× TAE buffer containing the same concentration of Na^+ and K^+ ions. The annealed DNA, RNA, or RNA:DNA samples were mixed with 1 µl of 10× loading dye containing bromophenol blue and glycerol before loading in the gels. The gels were stained using 0.5× aqueous GelRed (Biotium) solution for 20 min and destained in water for 10 min. The gels were imaged using Bio-Rad Gel Doc XR+ imager with the default settings for GelRed under ultraviolet illumination. Gels were analyzed using ImageLab.

Circular dichroism (CD) spectroscopy

The RNA complexes (10 µM) were prepared in 1× TAE buffer with 0-10 mM Mg^{2+} and annealed as described above. The CD spectra of the RNA solutions (200 µl) were recorded from 200 nm to 360 nm in a quartz cuvette of 1 mm thickness on a Jasco J-815 CD spectrometer with a scan speed of 100 nm/min, bandwidth of 1 nm, and digital integration time was set at 1 s. The data shown are the average of three accumulations.

Thermal melting studies

RNA complexes with a final strand concentration of 8 µM were annealed in 1× TAE buffer containing 0-10 mM magnesium acetate, 0-20 mM NaCl, or 0-20 mM KCl. Absorbance at 260 nm was recorded when the solutions were heated from 15 °C to 90 °C at a rate of 0.5 °C/min on a Cary 3500 UV-Visible Spectrophotometer (Agilent). The UV-thermal melting data were normalized, and the first derivative of the melting curves yielded the melting temperature (Tm).

Fluorescence spectroscopy

The fluorescence emission spectra of RNA complexes with ethidium bromide were recorded on a PerkinElmer Envision multimode plate reader. The emission spectra of RNA complexes with thiazole orange (TO) were recorded on a HORIBA Jobin Yvon Fluorolog-3-22 spectrofluorometer with a scan speed of 100 nm/min. The samples were annealed in 1× TAE buffer with the required amount of Mg^{2+}, as described above. Switchback RNA or conventional RNA duplex (at 2.5 μM) were incubated with different concentrations of the dye at 20 °C for 2 hours before the measurement. The samples containing EBr were excited at 520 nm, and the emission was recorded from 540 to 650 nm with 900 flashes. The enhanced fluorescence at 600 nm ($I-I_0$) was calculated as the difference between the intensity of emission in the sample with (I) and without RNA (I_0). The samples with thiazole orange were excited at 514 nm, and the emission spectra were recorded from 520 to 650 nm. The enhanced fluorescence at 530 nm was calculated as described above.

Enzyme degradation studies

Switchback RNA or duplex RNA were mixed with RNase III reaction buffer (final 1×) and $MnCl_2$ (final 20 mM) followed by enzyme addition. Enzyme dilutions were prepared in nuclease-free water. For the nuclease degradation assay, 1 μl of the RNase III dilution was added to 9 μl of the RNA/reaction buffer mixture and incubated at 37 °C for 30 min. For RNase H assay, 1 μl of the enzyme dilution was added to 9 μl of the mixture containing RNA-DNA hybrid sample and RNase H reaction buffer (final 1×) and incubated at 37 °C for 30 min. Samples were quenched in ice after incubation and loaded quickly onto the gels for electrophoresis.

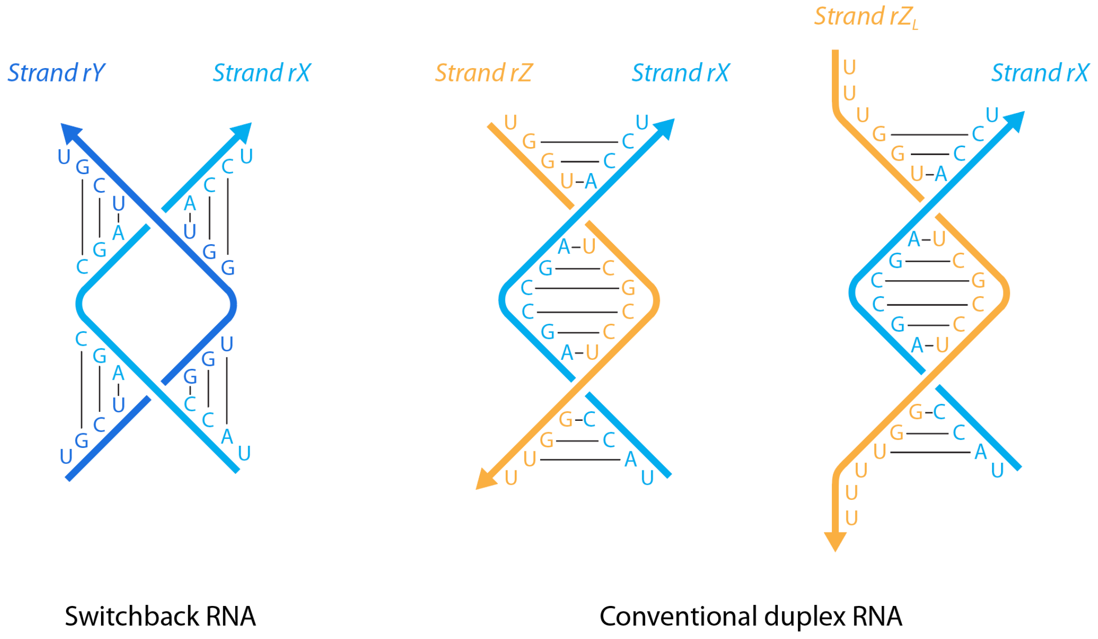

Figure S1. Design of switchback RNA and conventional duplex controls.

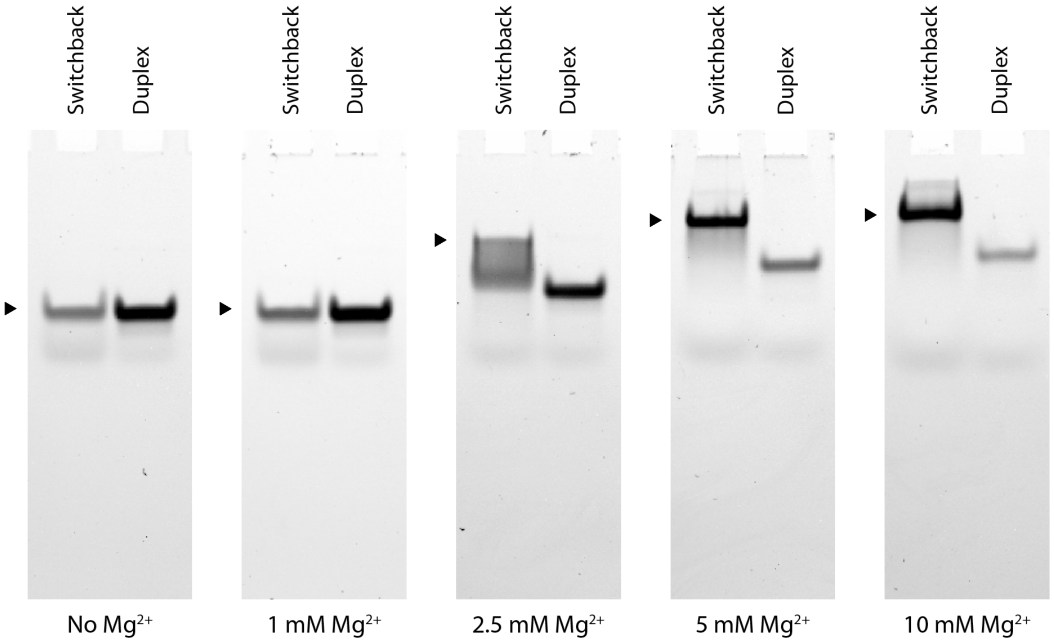

Figure S2. Non-denaturing gel images of switchback RNA and conventional duplex prepared in TAE buffer containing different concentrations of Mg^{2+}. The electrophoresis running buffers contained the same Mg^{2+} concentration as the respective annealing buffer.

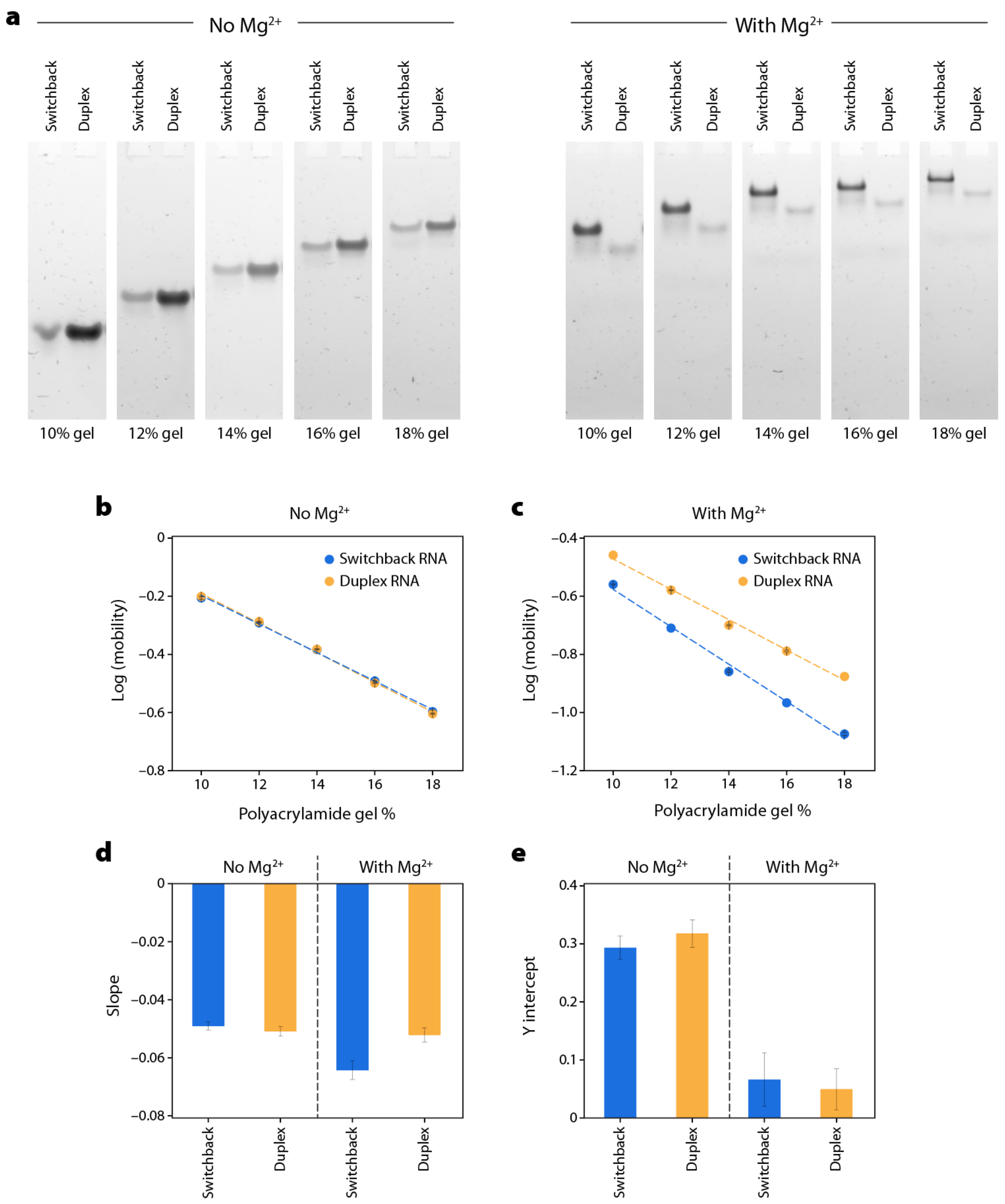

Figure S3. (a) Representative non-denaturing gels of switchback RNA and conventional duplex prepared without Mg^{2+} in the buffer and with 12.5 mM Mg^{2+} in the buffer. Electrophoresis was performed on the gels with different acrylamide concentrations (10-18%) using TAE and TAE-Mg^{2+} buffers, respectively. (b) Ferguson plots of switchback RNA and conventional RNA duplex in Mg^{2+}-free TAE buffer. (c) Ferguson plots of switchback RNA and conventional RNA duplex in TAE-Mg^{2+} buffer. (d) Comparison of the slope derived from the Ferguson plots of switchback RNA and conventional duplex. (e) Comparison of the Y-intercepts of the Ferguson plots. Data in (b) and (c) represent mean and standard deviations of experiments performed in duplicates. Error bars in (d) and (e) are standard errors from fitting the data.

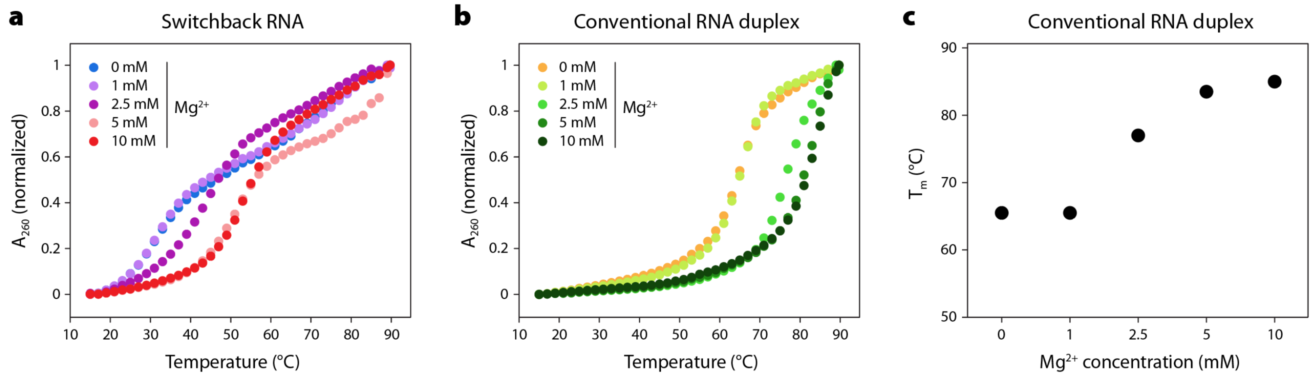

Figure S4. (a) Melting curves of switchback RNA at different Mg^{2+} concentrations. (b) Melting curves of conventional RNA duplex at different Mg^{2+} concentrations. (c) Melting temperatures of the conventional RNA duplex at different Mg^{2+} concentrations.

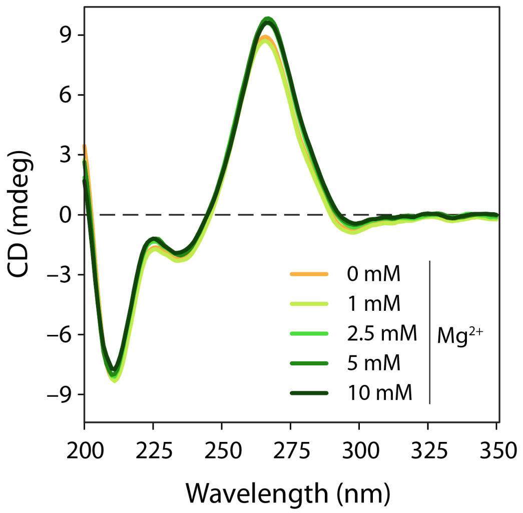

Figure S5. Circular dichroism spectra of conventional RNA duplex (rX·rZ) at different Mg^{2+} concentrations.

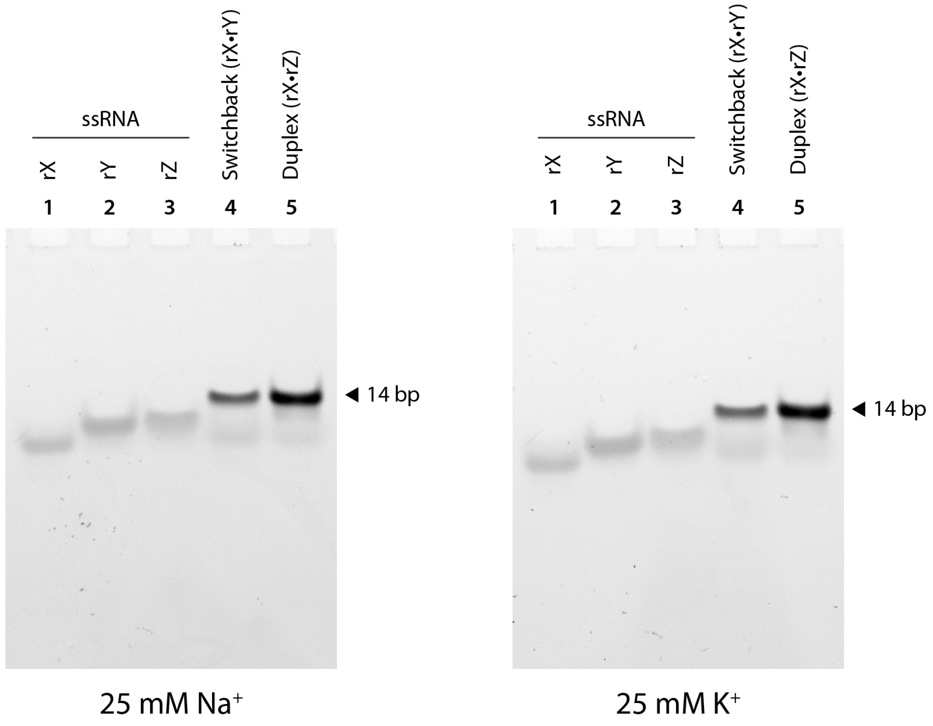

Figure S6. Non-denaturing gel image showing the assembly of switchback RNA in TAE buffer containing 25 mM NaCl and 25 mM KCl.

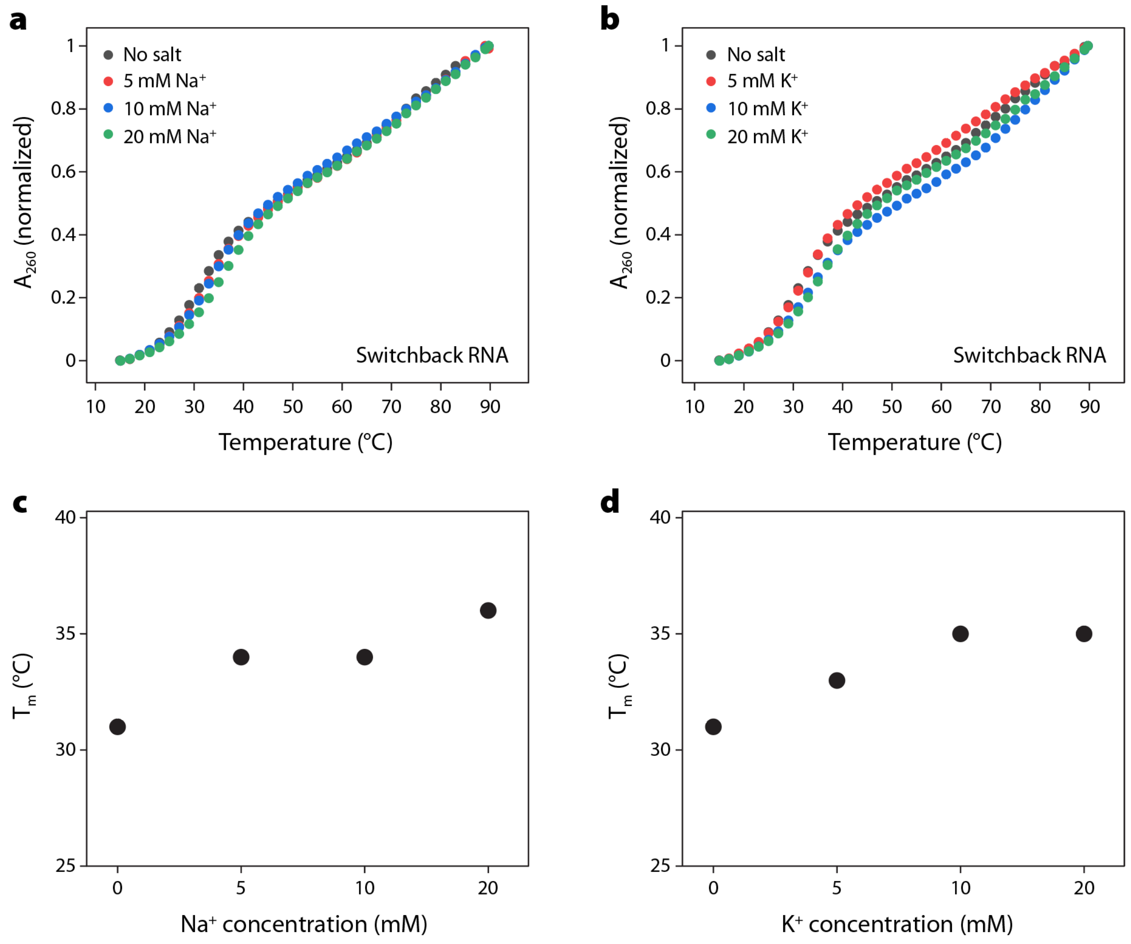

Figure S7. (a) Melting curves of switchback RNA prepared in TAE buffer with different concentrations of Na^+. (b) Melting curves of switchback RNA duplex prepared in TAE buffer with different concentrations of K^+. (c) Effect of Na^+ concentration on the melting temperature of switchback RNA. (d) Effect of K^+ concentration on the melting temperature of switchback RNA.

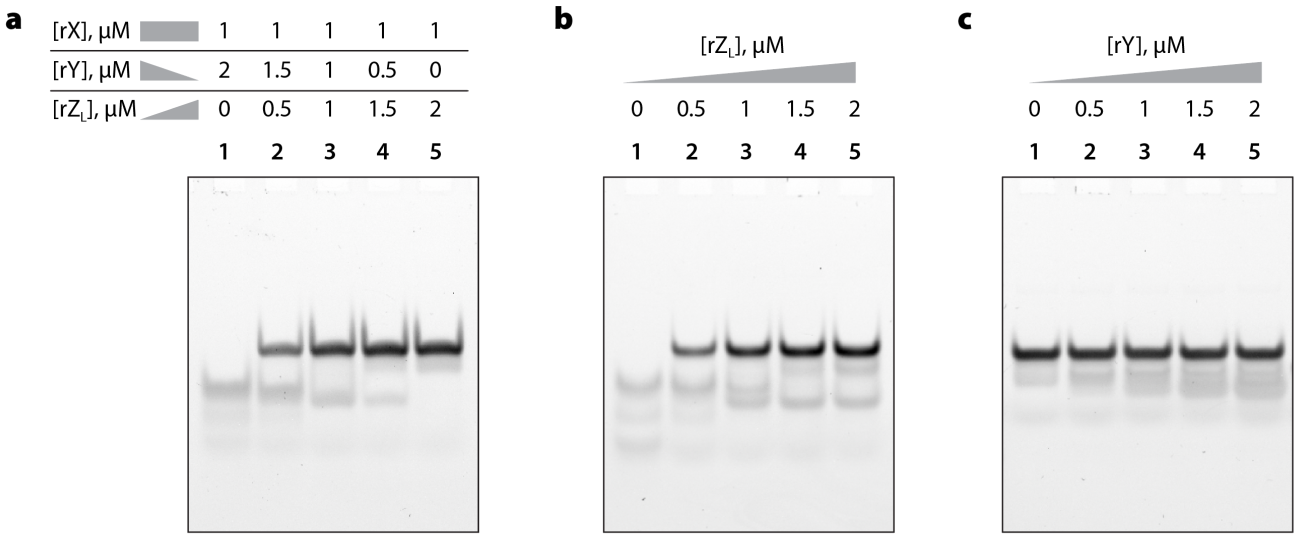

Figure S8. (a) Non-denaturing gel showing the strand competition between rY and rZ_L to bind to rX. (b) Toehold-less strand displacement leading to conversion of switchback RNA to conventional duplex. (c) Effect of excess switchback complement on conventional duplex. (a-c) are full images of gels shown in Figure 2.

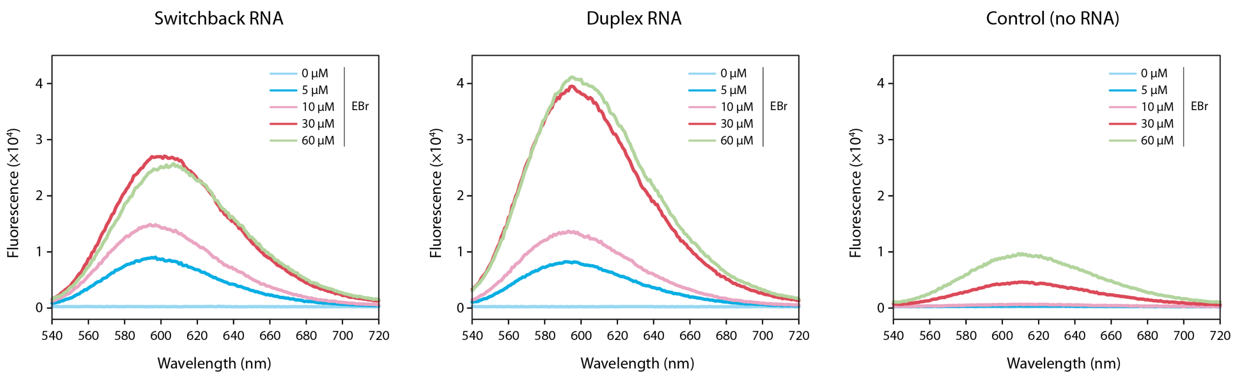

Figure S9. Fluorescence emission spectra of 0 to 60 μM ethidium bromide (EBr) in the presence of switchback RNA, conventional RNA duplex, and only 1× TAE buffer. The concentration of RNA complexes in the solutions was 2.5 μM.

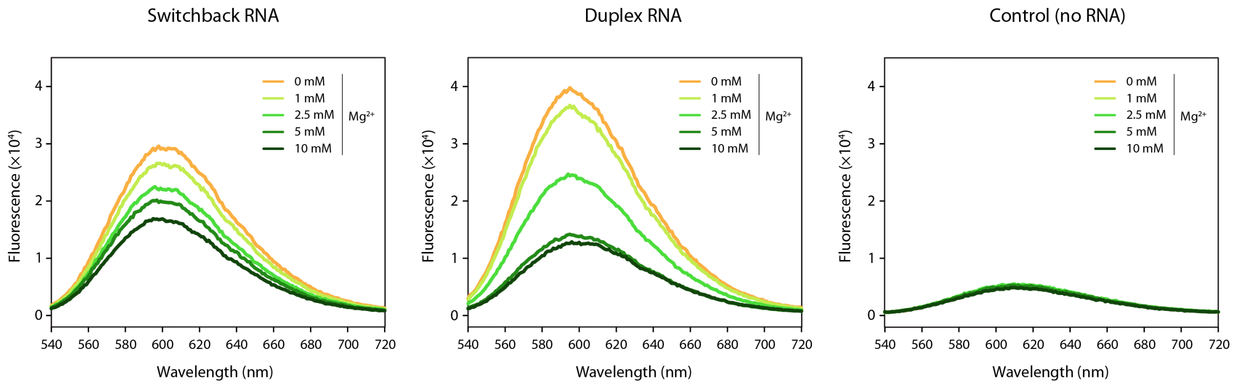

Figure S10. Fluorescence emission spectra of ethidium bromide (EBr) upon binding to switchback RNA, conventional RNA duplex and 1× TAE buffer containing 0 to 10 mM concentration of Mg^{2+}. The concentration of EBr was 30 μM, and the concentration of RNA strands in each solution was 5 μM.

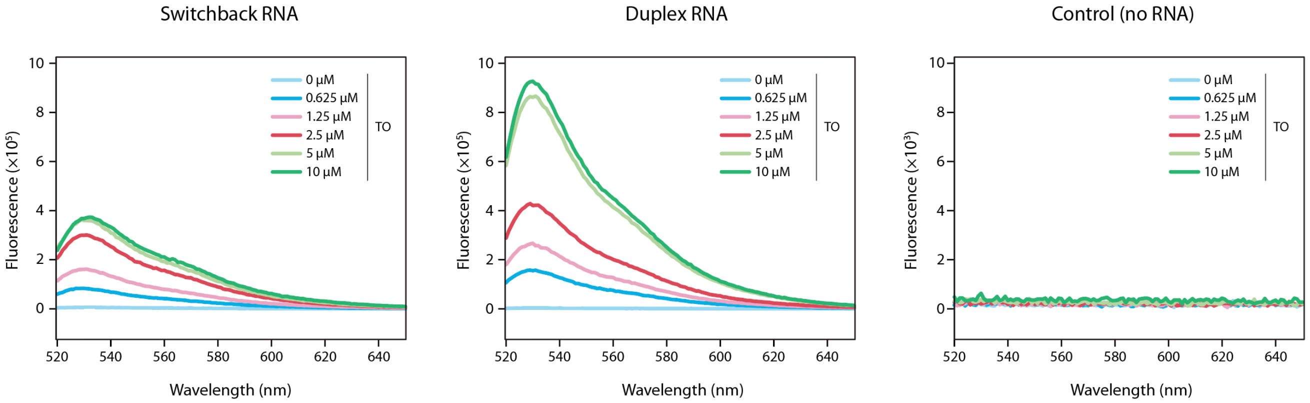

Figure S11. Fluorescence emission spectra of 0 to 10 μM thiazole orange (TO) in the presence of switchback RNA, conventional RNA duplex, and only 1× TAE buffer. The concentration of RNA complexes in the solutions was 2.5 μM.

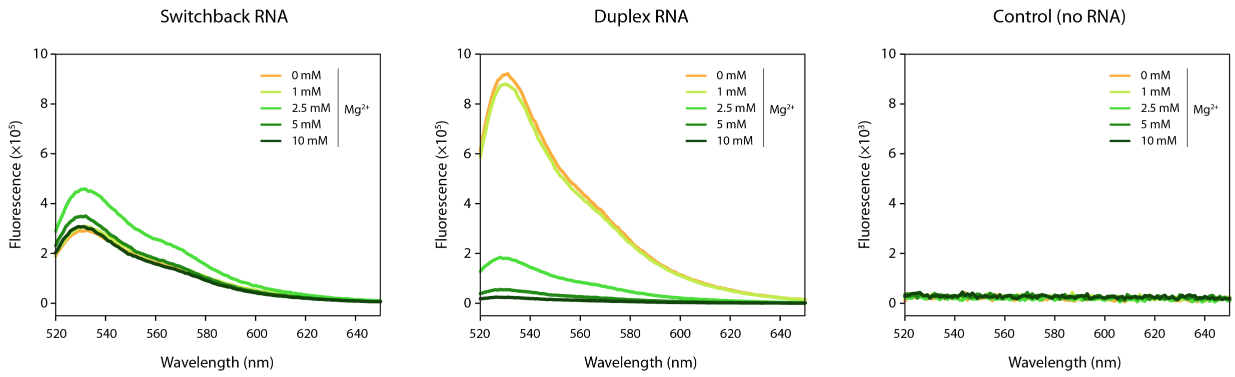

Figure S12. Fluorescence emission spectra of thiazole orange (TO) upon binding to switchback RNA, conventional RNA duplex and 1× TAE buffer containing 0 to 10 mM concentration of Mg^{2+}. The concentration of TO was 5 μM, and the concentration of RNA complexes in the solutions was 2.5 μM.

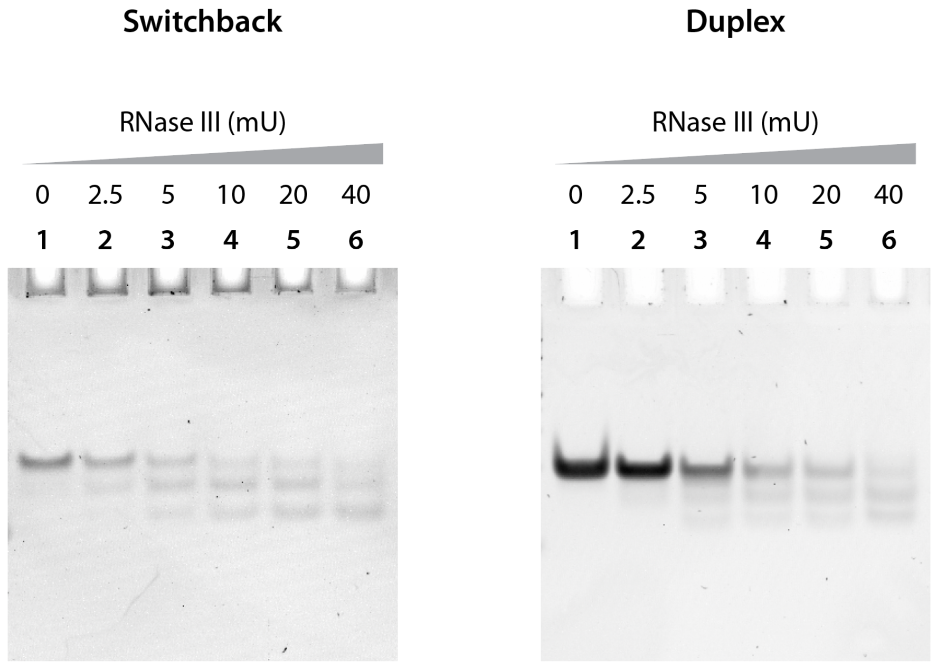

Figure S13. Non-denaturing gels showing switchback RNA and conventional duplex treated with RNase III. Full images of gels shown in Figure 4a.

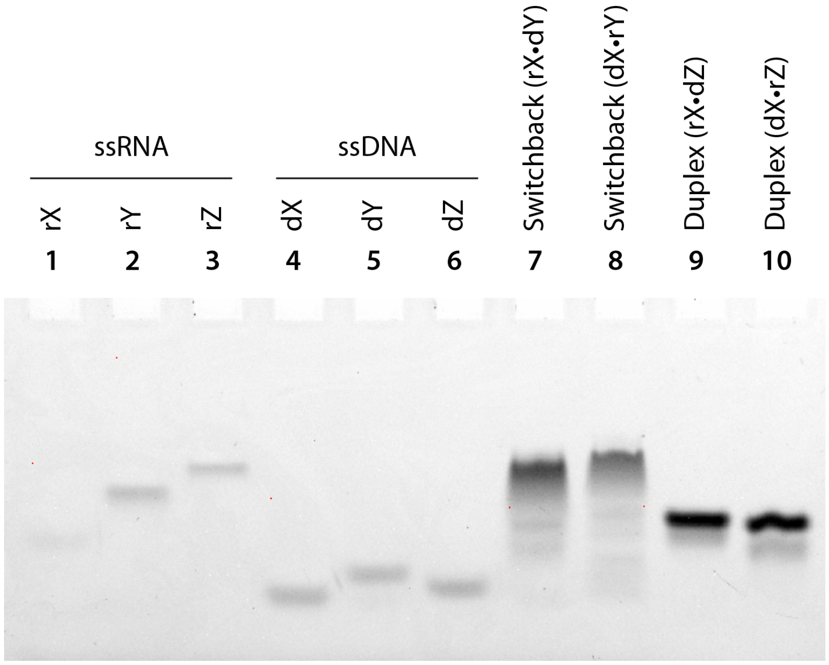

Figure S14. Assembly of switchback RNA:DNA hybrids.

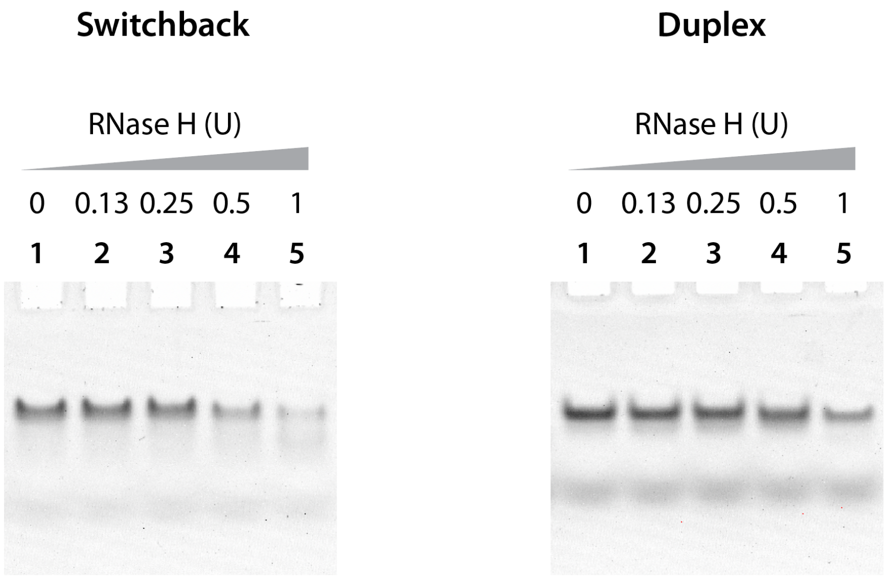

Figure S15. Non-denaturing gels showing switchback and duplex RNA:DNA hybrids treated with RNase H. Full images of gels shown in Figure 4b.

Table S1. Sequences used in this study.

Name	Sequence (5′-3′)	Length
RNA-rX	UACCAGCCGAACCU	14
RNA-rY	UGCUGGUGGUUCGU	14
RNA-rZ	UGGUUCGGCUGGUU	14
RNA-rZ_L	UUUGGUUCGGCUGGUUUU	18
DNA-dX	TACCAGCCGAACCT	14
DNA-dY	TGCTGGTGGTTCGT	14
DNA-dZ	TGGTTCGGCTGGTT	14

Table S2. UV-melting temperatures of switchback RNA and conventional RNA duplex in TAE-Mg^{2+}.

Mg^{2+} concentration	Switchback RNA	Conventional RNA Duplex
0 mM	31 °C	65.5 °C
1 mM	31.5 °C	65.5 °C
2.5 mM	45.5 °C	77 °C
5 mM	52.5 °C	83.5 °C
10 mM	52 °C	85 °C

Table S3. UV-melting temperatures of switchback RNA in TAE-Na^+ and TAE-K^+ buffers.

Ion concentration	Na^+	K^+
5 mM	34 °C	33 °C
10 mM	34 °C	35 °C
20 mM	36 °C	35 °C
